# Insights from the shotgun-based proteomics analysis of Biomphalaria glabrata

**DOI:** 10.6026/97320630017266

**Published:** 2021-01-31

**Authors:** Benson Otarigho, Mofolusho Falade, Castro-Borges William

**Affiliations:** 1 Department of Molecular Microbiology and Immunology, Oregon Health and Science University, OR, Portland, USA; 2 Department of Biological Science, Edo University, Iyamho, Nigeria; 3 Cellular Parasitology Programme, Cell Biology and Genetics Unit, Department of Zoology, University of Ibadan, Ibadan, Nigeria; 4 University of Kentucky College of Pharmacy, Lexington, KY, USA; 5 Departamento de Ciencias Biologicas, Núcleo de Pesquisas em Ciências Biologicas, Universidade Federal de Ouro Preto, Ouro Preto, Brazil

**Keywords:** Shotgun proteomics, Biomphalaria glabrata, hemolymph, Schistosomiasis, Bioinformatics

## Abstract

Biomphalaria glabrata is an important host in the transmission of human schistosomiasis in the Caribbean and South America. Therefore, it is of interest to analyse the proteome data of Biomphalaria glabrata hemolymph to identify immunity related proteins in host-pathogen relationship. We used shotgun proteomic and bioinformatic analyses of the non-depleted and depleted [0.5 and 0.75% Trifluoroacetic acid (TFA) depletion] hemolymph of B. glabrata (LE strain). Analysis showed 148 proteins from the hemolymph. 148 were obtained from the 0.5% TFA-depleted sample. 62 proteins follow this from the 0.75% TFA-depleted sample. However, only 59 were found from non-depleted hemolymph. A number of proteins were identified from the hemolymph of this schistosomiasis snail vector linked to immunity related functions. This provides insights to the understanding of schistosome-snail interaction.

## Background:

Biomphalaria glabrata is an important host in the transmission of human schistosomiasis [1,2], which has been reported in 78 countries, amounting to more than 240 million people infected worldwide [3]. Although control measures involving the combined use of molluscicides and mass chemotherapy have been in place, the disease burden is still high and spreading especially in the developing countries [4,5]. The knowledge on proteome composition of B. glabrata hemolymph, in which the internal defense mechanisms are impended, could provide clues in vaccine development. Proteomic analysis of Biomphalaria glabrata hemocytes have being shown [2] however, the proteome of the hemolymph is still unknow. Currently, advanced proteomic approaches have made it possible to identify proteins with greater ease and sensitivity [6,7]. In particular, the protein profile of B. glabrata hemolymph is dominated mostly by different proteoforms of hemocyanin [8]. Therefore, it is of interest to analyze the proteome data of Biomphalaria glabrata hemolymph to identify immunity related proteins in host-pathogen relationship.

## Materials and Methods: 

### Snails and hemolymph preparation:

B. Glabrata snails of the LE albino strain were used in this study. These snails were maintained in continuous culture in 40-liter aquarium which receive constant filtration and aeration at a constant temperature of 25°C with a 12: 12-hr light: dark cycle and were fed with fresh leaf lettuce ad libitum. Hemolymph was obtained from healthy B. glabrata snails (12–15 mm shell diameter) by the head-foot retraction method [9] and immediately placed on ice. Upon collection, hemolymph of each strain was dispensed into 1.5 mL microcentrifuge tubes containing cold, sterile Phosphate buffered saline (PBS) creating 1:1 dilution of PBS: hemolymph. 50ml of each sample was collected and check for the presence and absence of hemocytes before and after filtration respectively. The filtration was carried by passing each sample through 0.22um hydrophilic cellulose acetate filter membrane (EMD Millipore) by means of gentle press applied by an attached 5ml syringe. The filtrate was collected in 1.5ml microtube followed by addition of protease inhibitors (Protease Inhibitor Cocktail Set III, EDTA-free; Calbiochem, San Diego, CA) to protect proteins from endogenous protease activities.

### Cytospinning, hemocyte fixation and staining:

Cytocentrifuge was prepared with a labelled slide from the filtered hemolymph, chambered and blotted for each sample to be examined. 30ul of each sample was collected and diluted 1:1 in PBS and this was added to the slide chamber and spinned at 1500 rpm for 10 mins. Slides were carefully removed and allow to air dry prior to staining.

### Depletion of most abundant proteins:

To deplete hemocynin and other most abundant proteins from the hemolymph 5.3ul of prepared 10% trifluoroacetic acid (TFA) was added to 100ul of the hemolymph to have 0.5% depletion of most abundant protein while 8.1 ul of prepared 10% trifluoroacetic acid (TFA) was added to 100ul of the hemolymph to have 0.75% depletion of most abundant protein. This was followed by a gentle vortex and centrifuge at 2000g for 30mins at 4°C. The supernatant was collected and discard the pellet.

### 1D SDS-PAGE:

To evaluate and visualize the effect of TFA depletion of the most abundant proteins from this snail hemolymph, 1D SDS-PAGE were carried out on 12% gel at constant 20 mA in a Miniprotean 2D-chamber (BioRad). Supernatants obtained after depletion were mixed in a ratio of 1: 1 with loading buffer and under reducing conditions were heated at 95°C for 5 min prior to loading onto the gels. Briefly, 1st lane was loaded with 3.76 mg/ml of non-depleted hemolymph, 2nd lane was loaded with 2.32mg/ml of 0.75% TFA depleted hemolymph and 3rd lane was loaded with 2.03 mg/ml of 0.5% TFA depleted hemolymph. To facilitate comparisons, all samples were run simultaneously with low molecular weight markers (BioRad). After electrophoretic separation, the silver stained [10] (Shevchenko et al., 2000). The imaged of the gel was scanned on Image Scanner III (GE Healthcare) and exported using the labscan tools version 6.01. This analysis intended the evaluation of (1) the number of bands in each depletion compared to the non-depleted hemolymph and (2) to compare the huge bands at the beginning of the gel.

### In-solution digestion and Nanoflow UHPLC system in peptide/protein identification:

A total of 40ug of protein from each of the samples was digested with 1.6ug of sequencing grade trypsin. All samples were analyzed using liquid chromatography electrospray ionization tandem mass spectrometry (LC-ESI-MS/MS) on an UltimateTM 3000 series (Thermo Scientific DionexTM) coupled to a Q-Exactive mass spectrometer (Thermo Fisher Scientific, Bremen, Germany). The Q Exactive MS interfaced with nanoESI ionization as employed for MS analysis.

### B. Glabrata proteome and annotation:

The publicly available Proteome of Biomphalaria glabrata (BB02) version BglaB1.2 made available on 22th August, 2014, containing 14,137 protein sequences was downloaded from VectorBase, http://www.vectorbase.org, Biomphalaria glabrata, vector, BglaB1.2. And converted to fasta format using Geneious version R8 [11]. These sequences were functionally annotated on Blast2GO version 3.0 platform [12,13].

### Database searching and data analysis:

RawMeat software version 2.0 and Thermo Xcalibur 2.2 Qual Browser were employed to assess the quality of the chromatograms of each Thermo Xcalibur .Raw file and the efficiency of the protein digested with trypsin. All raw files were analyzed qualitatively and quantitatively using the Thermo Proteome Discoverer software platform version 1.4. The annotated proteome B. glabrata faste file was added to the platform fasta database and a label-free quantification with area detector and annotation workflow was used for the whole analysis. The analyzed results were filtered and exported in Excel workbook (xlsx format) where the enrichment graphs on scatter plot were plotted after proper filtration. The number of proteins in each depleted and non-depleted samples was identified on the server http://bioinformatics.psb.ugent.be/ webtools/Venn/ and the Venn diagram was generated on BioVenn (http://www.cmbi.ru.nl/cdd/biovenn/. The annotations generated by Blast2go on the identified proteins were manually confirmed on Gene Ontology database (http://amigo. geneontology.org/amigo/search/ontology). These protein annotations were plotted on a pie chart using the Excel software.

## Results:

**Figure 1:**
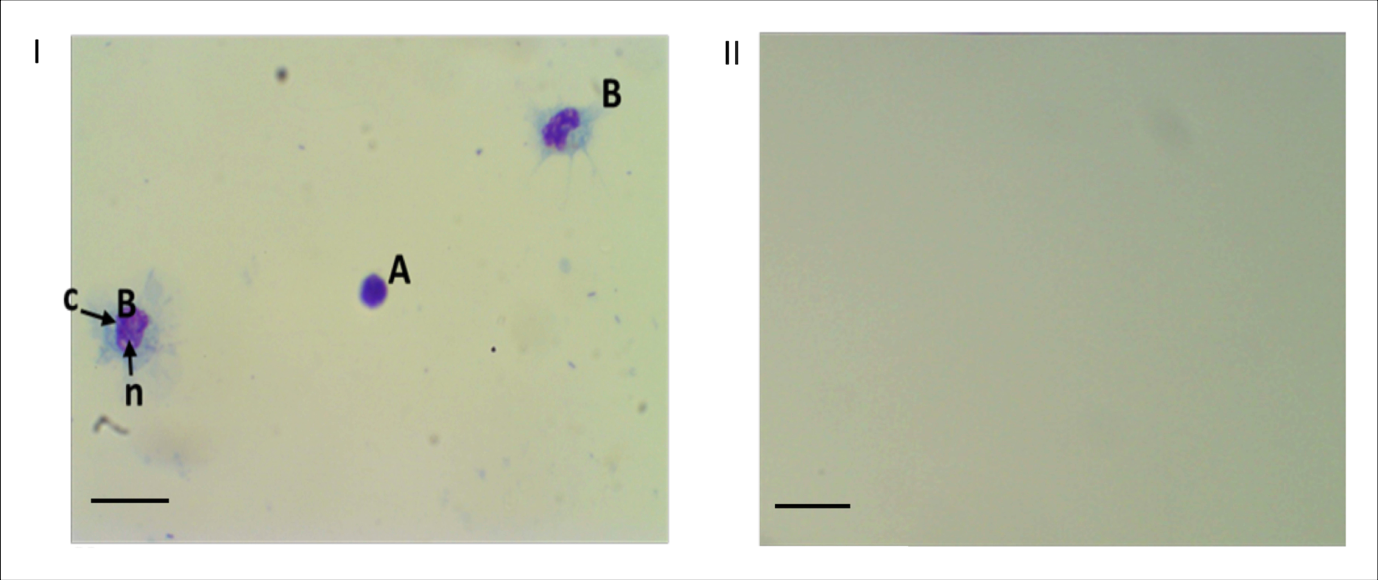
Stereomicroscope images showing the present or absence of hemocytes before and after filtration. I show present of hemocytes before filtration while II. Shows the absence of hemocyte after filtration. (A) Prohemocyte, (B) Oenocytoid, = (C) Cytoplasm, n = nucleus. Bar = 100µm

**Figure 2:**
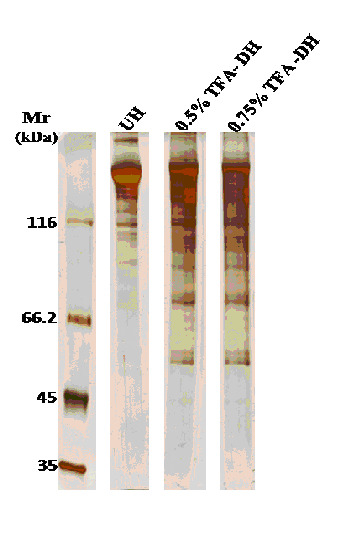
A comparative 1D gel analysis of non-depleted and TFA-depleted hemolymph revealed a significant enrichment for low molecular mass proteins (Mr < 116 kDa). 2µl of non-depleted hemolymph sample were loaded into 1st lane. 2.9µl of 0.5% TFA depleted sample were loaded into 2nd lane. Finally, 3.0µl of 0.75% TFA depleted sample were loaded into 3rd lane. *UH means non-depleted hemolymph; *DH means Depleted hemolymph

**Figure 4:**
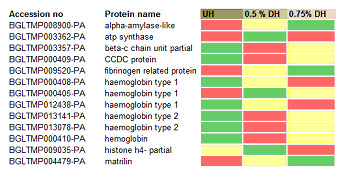
Heat map chart showing the quantitative effect of the depletion of 13 exclusive proteins in the three samples. The green, red and yellow cell show high, medium and low abundance respectively. *CCDC means coiled-coil domain-containing protein

The head-foot retraction method used in the hemolymph collection from these snails yielded about 500µl of hemolymph from snails. We observed that the slide-centrifuged method employed was able to remove the hemocytes and other cells that may be present from the hemolymph completely (Figure 1). This slide centrifuge method was to make sure that there were no cells or cell lysates in the samples before proceeding to depletion steps. The stereo microscopic images in Figure 1 shows the present of hemocytes before the samples were slide centrifuged while Figure 1 (ii) shows the absence of hemocytes after samples were slide centrifuged. We observed different stages of development of the hemocyte (prohemocyte and oenocytoid) in the hemolymph of B. glabrata as presented in Figure 1. The silver-stained 1D SDS PAGE gel in Figure 2 shows the comparative analysis of non-depleted and TFA-depleted hemolymph. The gel image revealed a significant enrichment for low molecular mass proteins (Mr < 116 kDa). In the non-depleted sample, there is a major huge dark band (Mr > 116 kDa) and a very few and faint bands are notice below 116kDa. In the depleted samples all bands show reproducible pattern. We noticed that there was a retained band in the stacking gel in the non-depleted sample, while it was not notice in the depleted samples. Again, between the two depleted samples, in the 0.5% TFA depleted sample, a band was noticed immediately the sample enter the separating gel but is absent in the 0.75% TFA depleted sample. However, the huge dark bands that are presented in non-depleted sample were highly reduced in depleted samples. Besides, there are much more proteins with lower molecular mass (Mr< 116 KDa) in the depleted samples when compare to non-depleted samples. The result show that all the data follow a non-parametric rule and the coefficient of variation across three replicates in non-depleted, 0.5% and 0.75% TFA depletion was 15.2%, 26.13 and 24.81% respectively. The lowest variation across replication was observed in the non-depleted sample. Upon interrogation of the LC-MS/MS results with a specific B. glabrata proteome dataset on proteome discoverer 1.4 platform, a total of 148 proteins groups were identified across the three samples, at a false discovery rate of 1-5%, when peptide confidence and protein per peptide were set at medium and 1 respectively. Since one of our primary aims is to have a comprehensive analysis of B. glabrata hemolymph proteome composition using shotgun approach, the number of identified proteins in each and shared by samples are represented by a Venn diagram in Figure 3A (see PDF version). A great number of proteins (95) were obtained from the 0.5% TFA-depleted sample, whilst 0.75% TFA-depleted and non-depleted hemolymph resulted in 61 and 58 identities, respectively. The unique number of proteins in 0.5% TFA depleted, 0.75% TFA depleted and non-depleted sample are 50, 18 and 37 respectively, while the unique number of proteins shared between 0.5% and non-depleted, 0.75% TFA depleted and non-depleted, 0.5% TFA and 0.75% TFA depleted sample are 5, 3 and 27 respectively. A total of 13 proteins were shared by the three samples. We constructed a heat map chart to quantitatively demonstrate the effect of TFA depletion on proteins that are common across the three and between the two depleted samples (Figure 4). It is clear that different proteoforms of hemocyanins, which are the most abundant proteins, were reduced in the depleted compared to non-depleted sample.

To have a meaningful interpretation of the identified proteins in terms of abundance and enrichment, we employed a scatter plot (Figure 3B - see PDF). From this scatter plot supported by the Venn diagram in Figure 3A (see PDF). We noticed that 0.5% TFA depletion was the better compare to 0.75% TFA depletion, since highest numbers of protein groups were identified. Most of these proteins were not identified in the non-depleted and 0.75% TFA depletion samples. From Figure 3A, the identified proteins in the group (i) were only in non-depleted sample and the protein group in (iii) were identified in 0.5% TFA depleted sample only. Proteins in group (ii) were identified in both non-depleted and 0.5% TFA depleted sample, however their abundant is higher or lower either in non-depleted hemolymph or 0.75% TFA depleted sample. The comparative list of proteins identified in both 0.5% TFA depleted and non-depleted samples. From Figure 3B (see PDF), the identified proteins in group (i) were only non-depleted sample and the protein in group (v) were identified in 0.75% TFA depleted sample only. Proteins in group (iv) were identified in both non-depleted and 0.75% TFA depleted sample; however there is some that there abundant is higher or lower either in non-depleted hemolymph or 0.75% TFA depleted sample. The comparative list of proteins identified in both 0.75% TFA depleted and non-depleted samples. Although, hemocyanin and it proteoforms were identified in all the three samples, their abundance in the depleted samples were much smaller in comparison to non-depleted samples. For us to have an idea of the possible activities of the identified proteins, we classified them bases on their molecular functions within gene ontology categories, which are represented (Figure 5 - see PDF). The majority (29%) of the identified proteins are enzymes, 11% are involved in transcription, 9% are involve in stress response/redox activities, 9% are involve in signaling, 7% are involved in defense against pathogens, 4% are involve in transport and 1% are involved in metabolism. Functions other than the mentions ones were classified as other processes and took 13%. The protein without annotation was classified as unknown function.

## Discussion:

In general, biofluid and other tissues pose great challenge in the field of proteomics, due to masking of low abundance proteins by the high abundance ones [7]. Studied had shown that the B. glabrata hemolymph is very important since it house the circulating hemocytes and protein components which play an immunological role in the relationship with parasites such, as S. mansoni developing larval, at the cellular and molecular level [2, 11]. These circulating hemocytes and protein components dictate the susceptibility, resistivity of the snail against parasite invasion and survival [2,14]. However, identification of these proteins which are low in abundance from this snail hemolymph is a big challenge due to the high abundance of proteoforms of hemocyanin [2]. Besides, this will pose a persistent barrier in search for biomarkers of pathogen infection and host resistant [7,15,16]. Although earlier studied found vital proteins in the hemolymph of the snail [2,17-19], the findings in the present work show that the depleted hemolymph of B. glabrata was more efficient protein extraction.

## Conclusion:

A number of novel proteins were identified from the hemolymph of this schistosomiasis snail vector and most of them were proposed to be crucially involve involved in immunity and others with unknown function were identified. Our investigation opens new avenues for elucidation of the schistosome-snail interaction. Besides, based on the findings in this work and apply this shot proteomic workflow, the search for biomarkers of S. mansoni infection in this snail hemolymph is on the way.
